# Measuring impostor phenomenon among health sciences librarians

**DOI:** 10.5195/jmla.2019.644

**Published:** 2019-07-01

**Authors:** Jill Barr-Walker, Michelle B. Bass, Debra A. Werner, Liz Kellermeyer

**Affiliations:** ZSFG Library, University of California, San Francisco, San Francisco, CA, jillbarr@gmail.com; Countway Library of Medicine, Harvard Medical School, Boston, MA, michelle_bass@hms.harvard.edu; John Crerar Library, University of Chicago, Chicago, IL, dwerner@uchicago.edu; Tucker Medical Library, National Jewish Health, Denver, CO, kellermeyerl@njhealth.org

## Abstract

**Objective:**

Impostor phenomenon, also known as impostor syndrome, is the inability to internalize accomplishments while experiencing the fear of being exposed as a fraud. Previous work has examined impostor phenomenon among academic college and research librarians, but health sciences librarians, who are often asked to be experts in medical subject areas with minimal training or education in these areas, have not yet been studied. The aim of this study was to measure impostor phenomenon among health sciences librarians.

**Methods:**

A survey of 2,125 eligible Medical Library Association (MLA) members was taken from October to December 2017. The online survey featuring the Harvey Impostor Phenomenon scale, a validated measure of impostor phenomenon, was administered, and one-way analysis of variance (ANOVA) was used to examine relationships between impostor phenomenon scores and demographic variables.

**Results:**

A total of 703 participants completed the survey (33% response rate), and 14.5% of participants scored ≥42 on the Harvey scale, indicating possible impostor feelings. Gender, race, and library setting showed no associations, but having an educational background in the health sciences was associated with lower impostor scores. Age and years of experience were inversely correlated with impostor phenomenon, with younger and newer librarians demonstrating higher scores.

**Conclusions:**

One out of seven health sciences librarians in this study experienced impostor phenomenon, similar to previous findings for academic librarians. Librarians, managers, and MLA can work to recognize and address this issue by raising awareness, using early prevention methods, and supporting librarians who are younger and/or new to the profession.

## INTRODUCTION

Impostor phenomenon, also known as impostor syndrome or impostor experience, is defined as an internal feeling of not deserving personal success that has been rightfully achieved [1]. Despite external evidence of their achievements, those with impostor phenomenon persist in believing that they are frauds and have fooled anyone who thinks otherwise. It is estimated that 70% of the population has experienced some form of this phenomenon [2]. Impostor phenomenon can have serious adverse effects including anxiety, depression, lack of confidence, decreased job satisfaction and performance, and inability to achieve in the face of self-imposed unattainable goals, which can lead to burnout [3]. Although this phenomenon has been studied among the academic workforce, there is little research focusing on librarians. The goal of this study was to measure impostor phenomenon among health sciences librarians and provide recommendations to address this issue.

### What is impostor phenomenon?

Impostor phenomenon was first studied by Clance and Imes in 1978 in a population of business executives using the Clance Impostor Phenomenon scale [1]. Researchers have identified three factors that define the impostor phenomenon: (1) believing that one has fooled others into overestimating one’s own abilities; (2) attributing personal success to factors other than one’s ability or intelligence, such as luck, extra work, charisma, or an evaluator’s misjudgment; and (3) fearing exposure as an impostor [4]. Those who experience impostor phenomenon feel that they are not good enough to keep their jobs, that others will discover their shortcomings, or that they might be fired at any moment despite evidence that they are high-performing employees. Those who experience impostor phenomenon may have an inability to acknowledge their own role in their accomplishments and, therefore, attribute their success to luck [1].

### Who experiences impostor phenomenon?

Impostor phenomenon has been studied in a range of professional fields such as accounting, marketing, and secondary education [3]. While this phenomenon has not previously been studied among health sciences librarians, it has been researched in the fields of health sciences and academia.

In the health sciences, most impostor phenomenon research focuses on students and residents, with studies showing that 30%–44% of medical students and residents [5, 6] and 38%–70% of nursing students [7–9] experience impostor phenomenon. Among medical students and residents, some evidence points to associations between impostor phenomenon and burnout [10, 11]. Among physician assistants—the only population of clinical practitioners to be extensively studied—impostor phenomenon was found to correlate with the presence of depression, with declining impostor scores associated with more years of practice [12, 13].

In academic settings, previous research supports the claim that the culture of academia and its hallmarks of “scholarly isolation, aggressive competitiveness, disciplinary nationalism, a lack of mentoring and the valuation of product over process” [14] can foster feelings of impostor phenomenon [3, 4, 14]. Academic faculty members report feelings of impostor phenomenon when they evaluate their scholarly productivity, when they engage in comparisons with faculty colleagues, and when their expertise is questioned [14]. In one study, teaching faculty and graduate teaching assistants who reported higher impostor scores received lower student evaluations of their teaching performance, were less likely to encourage questions and ideas in class, and had fewer academic advisees [15].

Although impostor phenomenon occurs in both men and women [3], some research in the academic and health sciences fields suggests that it is more prevalent among women. Studies of graduate and professional students in physics and family medicine report significant gender differences in impostor scores, with women exhibiting higher scores [6, 16]. Several researchers have considered how race intersects with impostor phenomenon in academia, including how students and administrators of color might experience higher impostor scores as a result of structural racism and individual racial discrimination [17–20]. Recent studies on the links between racial identity and impostor phenomenon show strong relationships between depression, perceived discrimination, and impostor feelings among Black undergraduate students [18].

### Librarian reports of impostor phenomenon

In the only existing study to measure impostor phenomenon among librarians, Clark et al. used the Harvey Impostor Phenomenon scale to measure the phenomenon among 352 academic librarians in the United States [4]. They found that 1 in 8 librarians reported above-average impostor scores. No differences in impostor scores were found by race, gender, or employment classification; however, younger librarians and those with less experience reported higher rates of impostor phenomenon [4]. Despite the lack of scholarly publications on the topic, the importance of impostor phenomenon in librarianship is reflected by many opinion pieces, blog posts, and conference presentations written by librarians in a variety of roles [21–27]. In these personal reflections, librarians share observations of self-doubt, minimization of their accomplishments, and the importance of recognizing impostor phenomenon.

### Study objective

The present study replicated the methods of Clark et al. [4] to measure impostor phenomenon among health sciences librarians and compare results to the population of academic college and research librarians in that study. Many health sciences librarians do not have educational backgrounds in the health sciences or work experience as clinicians, yet they are expected to be experts in these fields; therefore, the authors hypothesized that impostor phenomenon would be more prevalent among health sciences librarians than among academic college and research librarians, who often have educational backgrounds and advanced degrees in their subject areas.

Among health sciences librarians, we predicted differences in impostor phenomenon prevalence based on the type of library setting. Because it is rare for librarians to hold a clinical degree or have experience as practitioners, we hypothesized that hospital librarians would have higher impostor scores than those working in academic health sciences libraries. The underlying assumption for these hypotheses was that having a lack of formal expertise in the subject area in which one specializes would increase the likelihood of impostor phenomenon.

## METHODS

### Survey instrument

We used REDCap, a secure, web-based application designed to support data capture for research studies, to create and distribute an anonymous online survey [28]. Each survey had a unique link attached to each participant; however, as a security feature of REDCap, these links could not be viewed by the research team, thereby protecting the anonymity of participants. The survey included the Harvey Impostor Phenomenon Scale and seven demographic questions (supplemental Appendix A). We based several of the demographic questions on the Clark et al. survey in order to make comparisons with their results [4], and we used existing best practices to inform the creation of our questions about gender and racial identity [29, 30]. Based on feedback from study participants who expressed a desire to learn more about the study’s aims, we added a debriefing form at the end of the survey that included the study’s goals and links to additional resources.

### Harvey Impostor Phenomenon Scale

Harvey, a student of Clance, and Katz developed an alternative impostor phenomenon scale in 1981 [2]. Although the Harvey Impostor Phenomenon Scale and Clance Impostor Phenomenon Scale are both validated tools that have been widely used in recent research [31], we used the Harvey scale to allow comparisons with Clark et al.’s results [4]. The Harvey Impostor Phenomenon Scale is a list of 14 statements scored on a scale of 1 to 7, with the ends of the scale corresponding to “Not at all true” or “Very true”; some items are reverse-scored. For example, the statement, “At times, I have felt I am in my present position or academic program through some kind of mistake,” would be scored as 1 for “Not at all true” and 7 for “Very true,” whereas the statement, “I am certain my present level of achievement results from true ability,” would be scored as 1 for “Very true” and 7 for “Not at all true.” Total scores range from 0 to 84, with higher scores indicating a higher degree of impostor phenomenon. According to Harvey, a score of 42 or higher “may indicate possible troubles due to impostor feelings, and scores in the upper range suggest significant anxiety” [2].

### Data analysis

We cleaned data and created a codebook using Microsoft Excel and Google Docs (Appendixes B and C*) [32–34]. Univariate analysis and one-way analysis of variance (ANOVA) with Tukey’s post-hoc test were performed in Stata, a statistical analysis software, with all authors contributing to the evaluation of results using a collaborative, iterative process.

### Categorization of variables

To compare our study results with Clark et al.’s [4], we used the same variable categories in our survey instrument whenever possible. During analysis, we created new categories to accurately measure differences between populations. Because of the small number of respondents that selected an identity other than white (n=93, 13%), we categorized racial identities into 2 broad categories: white participants and participants of color. Additionally, in order to effectively compare differences among age groups, we collapsed Clark’s 10 age categories into 3 age categories, based on generational age groupings developed by the Pew Research Center [35].

### Participants

The study population was a census of Medical Library Association (MLA) members. Because we wanted to measure the experiences of practicing health sciences librarians in a US context, students, unemployed members, and members located outside of the United States were excluded. A total of 2,125 MLA members who fit this criterion were emailed in October 2017 with an invitation to complete an online survey, and 2 reminders were sent over the next 2 months. All data were collected between October and December 2017. This study was exempted from review by the University of California, San Francisco, Institutional Review Board (study #17-22873).

## RESULTS

A total of 703 participants completed the survey (33% response rate), with 57 additional incomplete responses that were not included in the analysis. Most participants were women (84%), white (84%), over 46 years old (59%), and had more than 11 years of experience in health sciences libraries (56%). Years of experience generally increased with age, indicating that librarians with more years of experience tended to be older. Most participants worked in academic health sciences libraries (57%) and did not hold any degrees in a health sciences discipline (79%). One in 7 health sciences librarians (14.5%) scored 42 or higher on the Harvey scale, indicating possible impostor feelings [2]. The mean and median impostor scores were 28.69 and 27, respectively, with a standard deviation of 11.3 and a range from 5 to 70. In comparison to the Clark et al. study, demographic characteristics of the 2 populations were similar, as were means, ranges, and percentages of impostor scores between groups (e.g., gender, race, age, and years of experience), with additional information presented in Table 1 and Appendixes D–I.

**Table 1:** Comparison of results between the present study and the Clark et al. 2014 study [4]

Present study (n=703)						Clark et al. study (n=352)					

	Minimum score	Maximum score	Mean score	Scores ≥42			Minimum score	Maximum score	Mean score	Scores ≥42	
	
				n	(%)					n	(%)
Gender						Gender					
Female (n=594)	5	70	28.61	85	(14%)	Female (n=262)	3	64	28.3	31	(12%)
Male (n=97)	6	58	28.8	16	(16%)	Male (n=90)	7	70	28.78	14	(16%)
Non-binary/third gender (n=3)	20	49	36	1	(33%)	N/A					
Prefer not to respond (n=9)	19	41	30.33	0	(—)	N/A					
Age (years)						Age (years)					
≤30 (n=46)	16	56	33.26	11	(24%)	≤30 (n=32)	7	70	35.34	8	(25%)
31–35 (n=76)	13	70	33.26	20	(26%)	31–35 (n=54)	7	63	30.48	11	(20%)
36–40 (n=86)	7	58	31.3	16	(19%)	36–40 (n=48)	8	59	27.92	8	(17%)
41–50 (n=166)	8	65	29.52	25	(15%)	41–50 (n=67)	4	57	29.07	7	(10%)
51–60 (n=166)	5	58	27.27	19	(11%)	51–60 (n=97)	3	58	26.81	8	(8%)
≥61 (n=163)	6	51	24.49	11	(7%)	≥61 (n=54)	4	64	24.8	3	(6%)
Years of experience						Years of experience					
<3 (n=80)	12	58	33.1	22	(28%)	<3 (n=48)	7	70	35.21	14	(29%)
3–6 (n=111)	13	70	33.29	26	(23%)	3–6 (n=49)	4	60	29.94	8	(16%)
7–10 (n=114)	7	66	29.91	19	(17%)	6–10 (n=53)	7	52	26.02	4	(8%)
11–20 (n=185)	5	58	27.04	17	(9%)	10–20 (n=90)	7	58	28.53	12	(13%)
>20 (n=213)	6	56	25.4	18	(8%)	>20 (n=112)	3	64	25.9	7	(6%)

One-way between-subjects ANOVA showed no significant effect of gender on impostor score [*F*(3, 699)=0.49, *p*=0.68]. Likewise, there was no significant difference in impostor score depending on racial identity categories [*F*(7,695)=0.59, *p*=0.76]. Librarians reporting a health sciences educational background (n=152) had mean and median scores of 26.74 and 24, respectively, with a standard deviation of 11.23 and a range from 7 to 65. Librarians who did not report a health sciences educational background (n=551) had mean and median scores of 29.22 and 24, with a standard deviation of 11.27 and a range from 5 to 70. We found a significant effect of reporting a health sciences educational background on impostor score [*F*(1, 701)=5.78, *p*=0.0165], with librarians who reported a health sciences educational background scoring slightly lower on the Harvey scale than those who did not. Contrary to our hypothesis, impostor scores of hospital librarians (n=230, M=27.55, SD=11.25) did not significantly differ from those of librarians who worked in academic health sciences settings (n=404, M=29.17, SD=11.40) [*F*(2, 700)=1.78, *p*=0.17].

**Figure 1:**
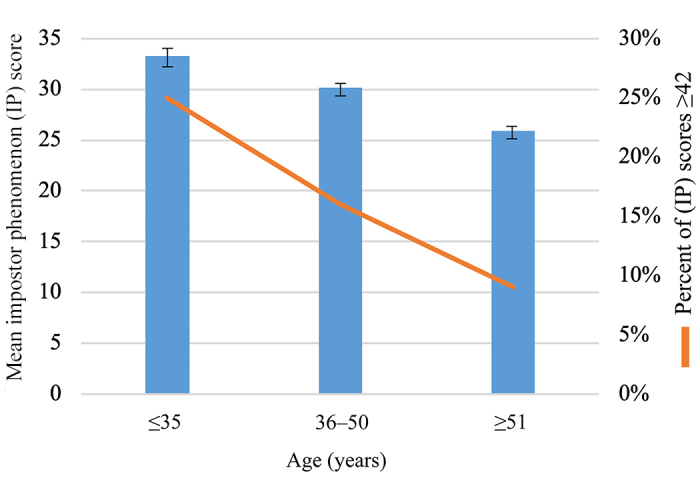
Comparison of impostor phenomenon scores by age

**Figure 2:**
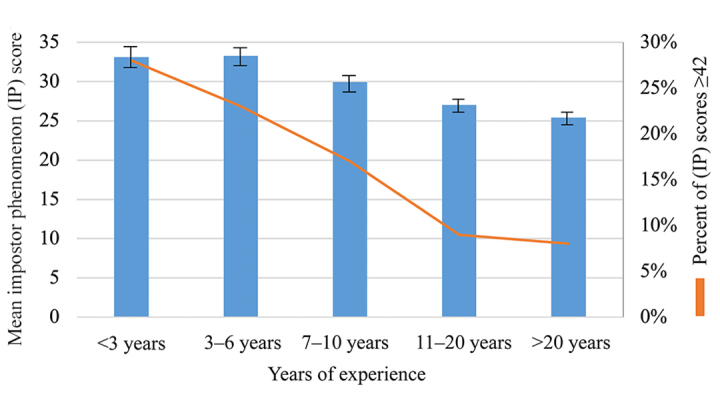
Comparison of differences in impostor scores by years of experience

One-way ANOVA with Tukey’s post hoc tests revealed significant associations between impostor phenomenon and 2 variables: age [*F*(2, 700)=23.57, *p*=0.0001] and years of experience [*F*(4, 698)=14.50, *p*=0.0001]. Examining differences between the collapsed age groups (≤35, 36–50, and ≥51 years), we found that impostor scores decreased as age increased (Figure 1, Appendix G). Post hoc comparisons using the Tukey honestly significant difference (HSD) test indicated that the mean score for the ≤35 age group (M=33.26, SD=11.40) was significantly higher than that for the ≥51 age group (M=25.89, SD=10.75) and that the mean score for the 36–50 age group (M=30.13, SD=11) was significantly higher than that for the ≥51 group. Impostor scores also decreased as years of experience increased. Post hoc comparisons using the Tukey HSD test indicated significant differences between the <3 years and 11–20 years (*p*<0.001) and >20 years (*p*<0.001) groups, the 3–6 years and 11–20 years (*p*<0.001) and >20 years (*p*<0.001) groups, and the 7–10 years and >20 years groups (*p*<0.05) (Figure 2, Appendix J).

## DISCUSSION

### Comparison between results of the present and Clark et al. studies

We expected to find that health sciences librarians’ impostor scores would be higher than those of the academic college and research librarians in the Clark et al. study [4], as academic librarians frequently have educational backgrounds and advanced degrees that align with their subject specialties, whereas health sciences librarians often do not. However, when comparing overall mean impostor scores between our study and the Clark et al. study, the scores were nearly identical. We also hypothesized that hospital librarians would score higher on the Harvey scale than those in other types of health sciences libraries as these librarians might not have clinical backgrounds; however, this difference was not found.

We did, however, find that librarians who reported a health sciences educational background had lower impostor scores than those who did not. Although our hypothesis that impostor scores would differ by type of library (e.g., academic, hospital) was not supported, there appeared to be some merit in understanding the intersection of a librarian’s subject responsibilities and their educational backgrounds. It is common practice for librarians without subject expertise to learn the intricacies of the subject on the job. Our results suggest there is room for improvement, and perhaps additional subject-related education would benefit those who experience stronger feelings of impostor phenomenon.

Additional findings in our study were consistent with those of the Clark et al. study, including significant differences in the means of impostor scores for years of experience. The similarities in these findings might be due to the fact that the majority of the respondents in the current study worked in academic settings and faced similar conditions as the academic librarians in the Clark et al. study. Future research comparing the impostor scores of librarians outside of academia—such as public librarians, special librarians, and nonacademic law librarians—might provide a broader picture of impostor phenomenon in librarianship.

### Considerations around race and gender

Previous research reports higher impostor phenomenon scores in people of color [17–20]. In our study, because only 13% of respondents selected a racial identity other than white, we grouped racial identities into 2 broad categories: white and people of color. In contrast to previous work, we found no differences in impostor scores in these racial identity categories; however, by grouping all people of color into a single category, any differences that might have existed between various racial identities were lost. Our small sample size for people of color is representative of the lack of diversity in health sciences librarianship [36], which in itself might be a factor in experiencing impostor phenomenon for librarians of color. One recent theory explores cultural and societal factors like race as causes of impostor phenomenon in libraries [37]. While additional empirical research is needed to explore a link to these cultural and societal factors, anecdotal evidence and research in other fields has pointed to associations between race and impostor phenomenon that should not be ignored [18]. Qualitative research exploring the experiences of librarians of color around impostor phenomenon can shed light on nuanced intersectional relationships between impostor phenomenon, race, gender, and privilege in the library workforce that have not been captured by existing research.

Some research shows that minority representatives in a group have higher impostor phenomenon scores, even if these are not traditionally oppressed groups in society (e.g., men faculty in women-dominated disciplines) [3]. In the current study, this was not the case: there were no significant differences in impostor scores between men and women, suggesting that the dynamics of male privilege might still be at work in the women-dominated field of librarianship. According to the most recent MLA salary survey, while 87% of the health sciences librarian workforce is made up of women, men in this field are paid up to 7.6% more than women in equivalent positions [36]. This difference cannot be explained by the commonly held belief that women are less likely to negotiate their salaries, as research shows that when librarians in non-administrative roles negotiate salary, women are less successful than men [38]. In light of increased attention to and scholarship on gendered roles and expectations of women librarians in recent years [39, 40], our findings can inform conversations around these issues.

### Implications for librarians and managers

Our results highlight disparities in impostor scores among newer and younger librarians, compared to librarians who are older or have more experience. As our results show, the more years of experience librarians have, the lower their impostor phenomenon score. But what, other than time, can help mitigate impostor feelings? McClurg and Jones list several interventions that employers can utilize, including low-stakes feedback and assessment early in one’s career, organizational socialization, strong workplace mentorship, continuing education, and affinity groups [41]. Those with supervisory or leadership roles in other fields have successfully addressed impostor phenomenon by modeling positive behaviors to their employees, such as delegating, demonstrating work-life balance, not overworking, giving praise, encouraging formal and informal mentoring opportunities, creating an environment where mistakes are accepted, and creating an organizational culture where communication is valued [4, 42, 43]. Clark et al. suggest that supervisors should recognize the signs of impostor phenomenon among their employees, such as when they minimize their own achievements or state they are not as good as others believe [4].

Early prevention is important, and supervisors should look for these signs—especially among new and younger staff members—to prevent burnout and low employee retention [42]. Raising awareness of impostor phenomenon by discussing it with colleagues and staff, perhaps even building it into new librarian orientations or partnering with library school events, can help eliminate stigma around impostor phenomenon and normalize the experience as something that has happened to almost everyone at some point in their working lives [14, 41].

### Future directions: the Medical Library Association’s role

The American Library Association has held several webinars focusing on impostor phenomenon, indicating recognition of and support for addressing this issue [37, 44]. MLA, as the professional association for health sciences librarians, could support its members by showing a similar commitment: a formal statement or meeting forum on the existence of impostor phenomenon, including how to recognize and combat these feelings, would be a logical starting point. Formats such as webinars or in-person panel discussions would allow participant interaction and input from a diverse group of voices. A permanent section on the MLA website could contain information about impostor phenomenon and link to other MLA resources designed to help new members, including networking or mentoring opportunities, information about paths to leadership positions, and groups with similar professional interests and goals, including local or chapter groups.

Mentorship could be a particular area of focus for MLA. In the current MLA mentoring system, the burden is on the interested mentee to establish communication with a mentor listed in a comprehensive directory. Because new and younger librarians experience feelings of impostor phenomenon at higher rates, a program designed to match dedicated mentors with new members may be helpful. In such a program, mentors could be trained and encouraged to discuss impostor phenomenon and strategies to combat these feelings if they arise. Mentors could elicit specific professional goals from mentees and gauge progress toward these goals throughout their first year. A structured program like this would provide dedicated allies and guides to the populations in our field that are most vulnerable to impostor phenomenon.

### Limitations

Selection bias may have been present in the study as the results might not represent health sciences librarians who did not belong to MLA, including those new to the profession or unable to fund membership. Because we did not include participants based outside of the United States, we could not draw conclusions about the experiences of impostor phenomenon among health sciences librarians on a global scale. However, the study population was chosen to closely mirror the population of the Clark et al. study [4], which included only US-based librarians. Despite these limitations, surveying MLA members remains the best way to capture the widest participation of health sciences librarians in a US context.

The respondents were self-selected; those who did not experience feelings of impostor phenomenon or inadequacy may have declined to respond. Conversely, those who identified closely with the questions in the survey or were reminded of negative experiences concerning impostor feelings may have chosen not to participate, and the experiences of these groups may have differed from those who completed the survey. We attempted to address these limitations by using the validated Harvey Impostor Phenomenon scale and using rigorous statistical methods to evaluate results.

## CONCLUSION

One in seven health sciences librarians in the study experienced impostor phenomenon, with younger and newer librarians showing higher impostor scores. Recognizing the signs of impostor phenomenon, raising awareness about its prevalence, and supporting librarians through mentoring, colleague support, and educational efforts are some of the ways that librarians, managers, MLA, and the larger profession can address impostor phenomenon. Future research involving comparisons of librarians inside and outside of academia may uncover trends and lead to collaborative solutions across librarianship. Additional qualitative research, including an exploration of the intersectional relationships between race, gender, and privilege in the library workforce is needed to further understand how health sciences librarians experience impostor phenomenon.

## SUPPLEMENTAL FILE

Appendix A Survey instrument

Appendixes B–J are accessible in an open repository hosted by the University of California (DASH), as noted in the “Data Availability Statement.”

## Footnotes

* Appendixes B–J are accessible in an open repository hosted by the University of California (DASH), as noted in the “Data Availability Statement.”

## References

[1] Clance PR, Imes SA (1978). The imposter phenomenon in high achieving women: dynamics and therapeutic intervention. Psychol Psychother Theor Res Pract.

[2] Harvey JC, Katz C (1985). If I’m so successful, why do I feel like a fake? the impostor phenomenon.

[3] Parkman A (2016). The imposter phenomenon in higher education: incidence and impact. J High Educ Theor Pract.

[4] Clark M, Vardeman K, Barba S (2014). Perceived inadequacy: a study of the imposter phenomenon among college and research librarians. Coll Res Libr.

[5] Henning K, Ey S, Shaw D (1998). Perfectionism, the impostor phenomenon and psychological adjustment in medical, dental, nursing and pharmacy students. Med Educ.

[6] Oriel K, Plane MB, Mundt M (2004). Family medicine residents and the impostor phenomenon. Fam Med.

[7] Aubeeluck A, Stacey G, Stupple EJ (2016). Do graduate entry nursing student’s experience ‘imposter phenomenon’?: an issue for debate. Nurs Educ Pract.

[8] Christensen M, Aubeeluck A, Fergusson D, Craft J, Knight J, Wirihana L, Stupple E (2016). Do student nurses experience imposter phenomenon? an international comparison of final year undergraduate nursing students readiness for registration. J Adv Nurs.

[9] Evan MM (1999). The impostor phenomenon: a descriptive study of its incidence among registered nurse preceptors [master’s thesis].

[10] Villwock JA, Sobin LB, Koester LA, Harris TM (2016). Impostor syndrome and burnout among American medical students: a pilot study. Int J Med Educ.

[11] Legassie J, Zibrowski EM, Goldszmidt MA (2008). Measuring resident well-being: impostorism and burnout syndrome in residency. J Gen Intern Med.

[12] Prata J, Gietzen JW (2007). The imposter phenomenon in physician assistant graduates. J Physician Assist Educ.

[13] Mattie C, Gietzen J, Davis S, Prata J (2008). The imposter phenomenon: self-assessment and competency to perform as a physician assistant in the United States. J Physician Assist Educ.

[14] Hutchins HM, Rainbolt H (2017). What triggers imposter phenomenon among academic faculty? a critical incident study exploring antecedents, coping, and development opportunities. Hum Resour Dev Int.

[15] Brems C, Baldwin MR, Davis L, Namyniuk L (1994). The imposter syndrome as related to teaching evaluations and advising relationships of university faculty members. J High Educ.

[16] Collett J, Avelis J Family-friendliness, fraudulence, and gendered academic career ambitions.

[17] Bernard DL, Hoggard LS, Neblett EW (2018). Racial discrimination, racial identity, and impostor phenomenon: a profile approach. Cult Divers Ethnic Minor Psychol.

[18] Cokley K, Smith L, Bernard D, Hurst A, Jackson S, Stone S, Awosogba O, Saucer C, Bailey M, Roberts D (2017). Impostor feelings as a moderator and mediator of the relationship between perceived discrimination and mental health among racial/ethnic minority college students. J Counsel Psychol.

[19] Moua G (2014). Impostor phenomenon among Hmong college students. Assess.

[20] Peteet BJ, Montgomery L, Weekes JC (2015). Predictors of imposter phenomenon among talented ethnic minority undergraduate students. J Negro Educ.

[21] Eckert C Privilege, intention and imposter syndrome. Storytime Underground [Internet].

[22] Faulkner AE (2015). Reflections on the impostor phenomenon as a newly qualified academic librarian. New Rev Acad Libr.

[23] Gordon RS (2003). Overcoming the systems librarian imposter syndrome. LIBRES.

[24] Hare J (2016). Becoming a librarian: from Sydney to Hong Kong via a LIS degree. Aus Library J.

[25] Lacey S, Parlette-Stewart M (2017). Jumping into the deep: imposter syndrome, defining success and the new librarian. Partnership: Can J Libr Inform Pract Res.

[26] Murphy B Imposter syndrome as a student. Hack Library School [Internet].

[27] Sobotka C Dealing with imposter syndrome and feeling like you belong. INALJ [Internet].

[28] Harris PA, Taylor R, Thielke R, Payne J, Gonzalez N, Conde JG (2009). Research electronic data capture (REDCap): a metadata-driven methodology and workflow process for providing translational research informatics support. J Biomed Inform.

[29] Consortium of Higher Education LGBT Resource Professionals (2015). Policy & practice recommendations [Internet].

[30] Johnston MP, Ozaki CC, Pizzolato JE, Chaudhari P (2014). Which box(es) do I check? investigating college students’ meanings behind racial identification. J Student Aff Res Pract.

[31] Holmes SW, Kertay L, Adamson LB, Holland CL, Clance PR (1993). Measuring the impostor phenomenon: a comparison of Clance’s IP Scale and Harvey’s IP Scale. J Pers Assess.

[32] Silverman D, Denzin NK, Lincoln YS (2003). Analyzing talk and text. Collecting and interpreting qualitative materials.

[33] Wolcott H, Conrad CF, Haworth JG, Lattuca LR (2001). Description, analysis, and interpretation in qualitative inquiry. Qualitative research in higher education: expanding perspectives.

[34] Connaway LS, Radford ML, Connaway LS, Radford ML (2017). Analysis of qualitative data. Research methods in library and information science.

[35] Dimock M Defining generations: where millennials end and generation Z begins [Internet].

[36] Corcoran K (2013). Medical Library Association MLA compensation and benefits survey.

[37] Conner-Gaten A, Van Ness S (2018). Battling impostor syndrome in the workplace [webinar].

[38] Silva E, Galbraith Q (2018). Salary negotiation patterns between women and men in academic libraries. Coll Res Libr.

[39] Emmelhainz C, Pappas E, Seale M, Accardi M (2017). Behavioral expectations for the mommy librarian: the successful reference transaction as emotional labor. The feminist reference desk.

[40] Lew S, Baharak Y (2017). Feminists among us: resistance and advocacy in library leadership.

[41] McClurg C, Jones R, Percell J, Sarin LC, Jaeger PT, Bertot JC (2018). Imposter phenomenon and the MLIS. Re-envisioning the MLS: perspectives on the future of library and information science education.

[42] de Vries MFK (2005). The dangers of feeling like a fake. Harv Bus Rev.

[43] Parkman A, Beard R (2008). Succession planning and the imposter phenomenon in higher education. Coll Univ Prof Assoc Hum Resour J.

[44] Puckett J (2018). Impostor syndrome in instruction librarians: impact and solutions [webinar].

