# Supplementary material for: Measuring impostor phenomenon among health sciences librarians
Source: J Med Libr Assoc. 2019 Jul 1;107(3):323–32. doi: 10.5195/jmla.2019.644 (PMC6579590; doi:10.5195/jmla.2019.644)
Supplement: Appendix A [file jmla-107-323-s001.pdf]

JMLA Journal of the Medical Library Association

SC

Supplemental content to
J Med Libr Assoc. Jul;107(3):dx.doi.org/10.5195/jmla.2019.644
www.jmla.mlanet.org
© Barr-Walker, Bass, Werner, Kellermeyer 2019

# Measuring impostor phenomenon among health sciences librarians

**Jill Barr-Walker; Michelle B. Bass; Debra A. Werner; Liz Kellermeyer**

## APPENDIX A

### Survey instrument

We are asking you to take part in a research study by the University of California, San Francisco (UCSF). Being in this study is optional.

If you choose to be in the study, you will complete a survey that will take 10–15 minutes to complete. This study seeks to explore health sciences librarians' attitudes toward their work. You can skip questions that you do not want to answer or stop the survey at any time. The survey is anonymous, and no one will be able to link your answers back to you. Please do not include your name or other information that could be used to identify you in the survey responses.

Contact Jill Barr-Walker at jill.barr-walker@ucsf.edu with questions. If you have questions or concerns about your rights as a research participant, you can call the UCSF Institutional Review Board at 415.476.1814.

For each statement below, check the box that best indicates how true of you the statement is. Responses range from 1=not at all true to 7=very true. Your first thoughts and impressions are most important here, so answer as quickly and honestly as possible.

| | Not at all true | Not true | Slightly not true | Neutral | Slightly true | True | Very true |
|---|---|---|---|---|---|---|---|
| 1. In general, people tend to believe I am more competent than I really am. | O | O | O | O | O | O | O |
| 2. I am certain my present level of achievement results from true ability. | O | O | O | O | O | O | O |
| 3. Sometimes I am afraid I will be discovered for who I really am. | O | O | O | O | O | O | O |
| 4. I find it easy to accept compliments about my competence. | O | O | O | O | O | O | O |
| 5. I feel I deserve whatever honors, recognition, or praise I receive. | O | O | O | O | O | O | O |
| 6. At times, I have felt I am in my present position or academic program through some kind of mistake. | O | O | O | O | O | O | O |
| 7. I feel confident that I will succeed in the future. | O | O | O | O | O | O | O |

SC
Supplemental content to
J Med Libr Assoc. Jul;107(3):dx.doi.org/10.5195/jmla.2019.644
www.jmla.mlanet.org
© Barr-Walker, Bass, Werner, Kellermeyer 2019

| | Not at all true | Not true | Slightly not true | Neutral | Slightly true | True | Very true |
|---|---|---|---|---|---|---|---|
| 8. I tend to feel like a phony. | O | O | O | O | O | O | O |
| 9. My personality or charm often makes a strong impression on people in authority. | O | O | O | O | O | O | O |
| 10. I consider my accomplishments adequate for this stage in my life. | O | O | O | O | O | O | O |
| 11. In discussions, if I disagree with my boss, a professor, or the person in charge, I speak out. | O | O | O | O | O | O | O |
| 12. I often achieve success on a project, report, or test when I have anticipated I would fail. | O | O | O | O | O | O | O |
| 13. I often feel I am concealing secrets about myself from others. | O | O | O | O | O | O | O |
| 14. My public and private self are the same person. | O | O | O | O | O | O | O |

15. Do you use any strategies to address feelings of inadequacy at work? If so, please describe.

---

16. If applicable, how effective are these strategies in addressing those feelings of inadequacy?

---

17. How often have you experienced any of the following related to your work in the past month?

| | Never | Once or twice a month | Once a week | More than twice a week | Every day |
|---|---|---|---|---|---|
| Anxiety | O | O | O | O | O |
| Burnout | O | O | O | O | O |
| Passion for your work | O | O | O | O | O |
| Demotivation | O | O | O | O | O |
| Feeling overworked | O | O | O | O | O |
| Pride | O | O | O | O | O |
| Procrastination | O | O | O | O | O |

JMLA Journal of the Medical Library Association

SC

Supplemental content to
J Med Libr Assoc. Jul;107(3):dx.doi.org/10.5195/jmla.2019.644
www.jmla.mlanet.org
© Barr-Walker, Bass, Werner, Kellermeyer 2019

| | Never | Once or twice a month | Once a week | More than twice a week | Every day |
|---|---|---|---|---|---|
| Satisfaction | O | O | O | O | O |
| Stress | O | O | O | O | O |
| Trouble delegating | O | O | O | O | O |

Please answer the following questions. Your responses will not be used to identify you.

1. How do you identify your gender?
o Female
o Male
o Non-binary/third gender
o Prefer to self describe
o Prefer not to respond
Please specify.

2. Which of these best describes your racial identity? Select all that apply.
o White/Caucasian
o African American/Black
o American Indian/Alaska Native
o Asian American/Asian
o Native Hawaiian/Pacific Islander
o Hispanic/Latino
o Middle Eastern/North African
o Other race not provided
o Prefer not to respond
Please specify.

3. What is your age?
o 25 and under
o 26–30
o 31–35
o 36–40
o 41–45
o 46–50
o 51–55
o 56–60
o 61–65
o Over 65

Supplemental content to
J Med Libr Assoc. Jul;107(3):dx.doi.org/10.5195/jmla.2019.644
www.jmla.mlanet.org
© Barr-Walker, Bass, Werner, Kellermeyer 2019

4. Do you have formal educational training in the health sciences field?
- Yes
- No

4a. Please select all health sciences degrees that you hold.
- Bachelor’s, clinical (e.g., BSN)
- Bachelor’s, nonclinical (e.g., human biology)
- Master’s, clinical (e.g., MSN, CNM)
- Master’s, nonclinical (e.g., MPH)
- Doctorate, clinical (e.g., MD/DO, DPT, DNP)
- Doctorate, nonclinical (e.g., PhD in epidemiology)
- Other
- I do not have a degree in a health sciences discipline.

Please specify.

5. Do you have a master’s degree in library/information science?
- Yes
- No

6. How many years of work experience in health sciences libraries do you have?
- Less than 3
- 3–6
- 7–10
- 11–20
- Over 20

7. What type of library do you work in?
- Academic
- Hospital
- Other

Please specify.

Thank you for participating in this study. The purpose of this research is to help us learn more about imposter phenomenon, also known as imposter syndrome or imposter experience, among health sciences librarians and how librarians address imposter phenomenon in their work. Previous work has shown that academic librarians experience differing levels of imposter phenomenon based on age and years of experience. We are interested in examining these effects among health sciences librarians.

We surveyed all members of the Medical Library Association in the United States. In this study, you were asked to complete the Harvey Imposter Phenomenon Scale, a validated measure of imposter phenomenon, and describe strategies that you use to address feelings of imposter phenomenon at work.

SC
Supplemental content to
J Med Libr Assoc. Jul;107(3):dx.doi.org/10.5195/jmla.2019.644
www.jmla.mlanet.org
© Barr-Walker, Bass, Werner, Kellermeyer 2019

The results from this study will help us raise awareness about the prevalence of imposter phenomenon among health sciences librarians and begin to create and disseminate ideas for addressing this phenomenon.

Your responses were immediately de-identified after completion of the survey, and no one will be able to link your answers back to you. The survey is hosted by REDCap, a secure, web-based application designed to support data capture for research studies.

If you have further questions about the study, please contact Jill Barr-Walker at jill.barr-walker@ucsf.edu. If you have questions or concerns about your rights as a research participant, you can call the UCSF Institutional Review Board at 415.476.1814.

Additional reading:

- Clark M, Vardeman K, Barba S. Perceived inadequacy: a study of the imposter phenomenon among college and research librarians. Coll Res Libr. 2014 May 1;75(3):255–71. <http://crl.acrl.org/index.php/crl/article/view/16362>.
- de Vries MFK. The dangers of feeling like a fake. Harv Bus Rev. 2005 Sep 1;83(9):108. <https://hbr.org/2005/09/the-dangers-of-feeling-like-a-fake>.
